# Towards AI-Based Strep Throat Detection and Interpretation for Remote Australian Indigenous Communities

**DOI:** 10.3390/s25185636

**Published:** 2025-09-10

**Authors:** Prasanna Asokan, Thanh Thu Truong, Duc Son Pham, Kit Yan Chan, Susannah Soon, Andrew Maiorana, Cate Hollingsworth

**Affiliations:** 1 School of Electrical Engineering, Computing and Mathematics Sciences, Curtin University, Perth, WA 6102, Australia; prasanna.asokan21@gmail.com (P.A.); henry.truong@curtin.edu.au (T.T.T.); dspham@ieee.org (D.S.P.); susannah.soon@curtin.edu.au (S.S.); 2 Curtin School of Allied Health, Curtin University, Perth, WA 6102, Australia; a.maiorana@curtin.edu.au; 3 Exercise Physiology Department, Fiona Stanley Hospital, Perth, WA 6102, Australia; 4 School of Molecular and Life Sciences, Curtin University, Perth, WA 6102, Australia; cate.hollingsworth@curtin.edu.au

**Keywords:** deep learning, transformer architecture, large language model, strep throat detection and interpretation, vision sensors

## Abstract

Streptococcus pharyngitis (strep throat) poses a significant health challenge in rural and remote Indigenous communities in Australia, where access to medical resources is limited. Delays in diagnosis and treatment increase the risk of serious complications, including acute rheumatic fever and rheumatic heart disease. This paper presents a proof-of-concept AI-based diagnostic model designed to support clinicians in underserved communities. The model combines a lightweight Swin Transformer–based image classifier with a BLIP-2-based explainable image annotation system. The classifier predicts strep throat from throat images, while the explainable model enhances transparency by identifying key clinical features such as tonsillar swelling, erythema, and exudate, with synthetic labels generated using GPT-4o-mini. The classifier achieves 97.1% accuracy and an ROC-AUC of 0.993, with an inference time of 13.8 ms and a model size of 28 million parameters; these results demonstrate suitability for deployment in resource-constrained settings. As a proof-of-concept, this work illustrates the potential of AI-assisted diagnostics to improve healthcare access and could benefit similar research efforts that support clinical decision-making in remote and underserved regions.

## 1. Introduction

Streptococcal pharyngitis (commonly referred to as “strep throat”) is a highly contagious bacterial infection that disproportionately affects Indigenous people living in remote regions in Australia, where access to healthcare is often limited [1]. If left untreated, strep throat can lead to serious complications, including acute rheumatic fever and rheumatic heart disease [2]. Despite its potential for causing serious harm, strep throat is highly treatable; early diagnosis and timely administration of antibiotics can effectively prevent these adverse outcomes. However, individuals living in remote communities face significant barriers to accessing appropriate healthcare. These barriers include social disadvantage, inadequate healthcare infrastructure, limited health literacy and awareness of the importance of early treatment, and a shortage of trained medical personnel [3]. In these settings, resource-constrained healthcare infrastructures are frequently unable to meet the specific needs of the population, resulting in preventive care and restricted access to treatment services.

Furthermore, rural doctors report a need for better-integrated programs to strengthen procedural skills [4]. Among Indigenous people, limited access to culturally appropriate healthcare, long travel distances, and fear of clinical diagnosis further hinder timely care [3]. Therefore, there is a need for scalable and accessible solutions. An AI-based model offers a promising avenue to support clinical decision-making; implementing AI in strep throat diagnosis has the potential to reduce the burden on overstretched healthcare providers and improve early detection and management of conditions in underserved rural and remote Indigenous communities.

The research presented in this paper provides both qualitative and quantitative analysis using an AI-based model to aid in diagnosing strep throat when strep throat images are captured by a version sensor [5]. Building on the research of Yoo et al. [6], which used a strep throat diagnostic classifier for binary classification of strep throat for throat images, we extend their methodology by benchmarking across a wider variety of model architectures, including Swin Transformer [7,8] and ViT-B/16 [9]. We attempt to enhance model performance to improve reliability in medical settings and minimize the model size, facilitating deployment in rural and remote Indigenous communities. Despite detecting physical signs of strep throat, the proposed AI-based model is integrated with an explainable model for visual analysis. This compliments the strep throat diagnostic classifier by providing interpretability and reasoning; the proposed model attempts to improve the potential for using AI in clinical support settings. Specifically, we utilize the BLIP-2 in a Transformer-based multimodal architecture, as detailed in Bootstrapping Language-Image Pre-training with Frozen Image Encoders and Large Language Models (LLMs) [10]. BLIP-2 employs a vision adapter that enables LLMs to process and interpret images; it provides a highly efficient and lightweight solution for image-based tasks while achieving near-state-of-the-art performance. This technique balances the efficiency and effectiveness of the proposed AI-based model and optimizes the model for both performance and computational feasibility.

The rest of this paper is organized as follows: Section 2 gives the background of strep throat diagnosis in rural and remote Indigenous communities, reviews traditional diagnostic methods for strep throat, and highlights their limitations; this section also reviews the current state-of-the-art methods for strep throat detection and highlights their advantages and limitations. Furthermore, it discusses the critical role of AI in this domain and the challenges that hinder its straightforward implementation in clinical practice. Section 3 discusses the proposed AI-based model for strep throat diagnosis in rural and remote Indigenous communities. It explains the design novelty and mechanism of action. Section 4 discusses how we validated the performance of various classifiers and explainable models in terms of detection accuracy, inference time, and model size; the validation performance is reported. A conclusion and some future deployment of the proposed AI-based model is given in Section 5.

## 2. Strep Throat Diagnosis in Rural and Remote Indigenous Communities

Group A Streptococcal (GAS) infections represent a significant public health concern in rural and remote communities, particularly in Indigenous Australian populations. These infections commonly present as strep throat, scarlet fever, impetigo, or cellulitis [11]. The burden of GAS-related illnesses, especially complications such as acute rheumatic fever, disproportionately impacts rural and remote Indigenous communities, contributing to ongoing health inequities. Epidemiological studies estimate that GAS accounts for approximately 20–30% of throat infections in children and 5–15% in adults [11].

If treatment is inadequate or delayed, Group A Streptococcal (GAS) infections can result in serious health complications [2]. Acute rheumatic fever (ARF) is an autoimmune reaction triggered by untreated throat infection and characterized by inflammation of the heart, joints, skin, and brain. Current Australian clinical guidelines recommend hospitalization for affected individuals. Recurrent episodes of ARF are common and substantially increase the risk of developing rheumatic heart disease (RHD), a severe chronic condition. RHD involves scarring and stiffening of the heart valves, impairing proper blood flow and leading to complications such as stroke, endocarditis, adverse pregnancy outcomes, and potentially fatal events. Between 2017 and 2021, 55% of new RHD diagnoses occurred in Indigenous Australians under the age of 25. Both ARF and RHD are largely preventable. Primordial prevention strategies include improving access to functional health infrastructure, promoting better hygiene, and reducing overcrowding. Primary prevention relies on timely and accurate diagnosis of GAS infections followed by appropriate treatment.

A recent study revealed that the prevalence of pharyngitis in communities is substantially higher than that observed in healthcare facilities; it highlighted the likelihood of the under-detection of Group A Streptococcal (GAS) infections [1]. At the population level, the incidence of pharyngitis in communities is approximately 7.3 times greater than the number of cases diagnosed in clinical settings. Several observations support this disparity. For instance, research on the recognition of impetigo in both community and clinical environments confirmed that skin infections tend to be normalized by healthcare practitioners when their burden is high [12]. Furthermore, multiple social determinants reduce healthcare-seeking behaviors due to stigma, limited access to primary healthcare, logistical barriers and costs, and fear and racism experiences. Missed opportunities for timely antibiotic intervention significantly undermine efforts to reduce the risk of subsequent ARF by up to 90%.

### 2.1. Conventional Diagnostic Methods for Streptococcal Pharyngitis

Streptococcal pharyngitis typically presents with several clinical signs, including white or yellow tonsillar exudate, cervical lymphadenopathy, and pharyngeal inflammation. These are likely to be accompanied by systemic symptoms such as fever, nausea, and abdominal pain. However, pharyngitis can also be caused by viral or fungal pathogens; they exhibit similar clinical features but require different treatment strategies. Therefore, accurately distinguishing between bacterial and non-bacterial causes of pharyngitis based solely on symptoms and physical examination is challenging.

To enhance diagnostic accuracy, clinicians frequently use the Modified Centor Score in Table 1, a clinical prediction rule designed to estimate the likelihood of a Group A Streptococcal (GAS) infection based on patient history and physical findings [13]. This scoring system is derived from logistic regression analysis of historical patient data and demonstrates specificities of 72.4% and 93.7% when three or four clinical criteria are present, respectively [14]. Although alternative clinical scoring systems such as McIsaac and FeverPAIN are also widely used, studies suggest that there is no significant difference in diagnostic performance among them.

For more definitive diagnosis, especially when clinical criteria are inconclusive, Rapid Antigen Detection Tests (RADTs) are employed. These point-of-care swab tests yield results within minutes and offer a specificity of approximately 97.0%. Although RADTs can be more effective, they are likely to be inconsistently accessible or affordable in all healthcare settings, particularly in remote or resource-limited environments. Throat cultures remain the gold standard for confirming GAS infections since they have high accuracy; however, the 24–48 h turnaround time can delay clinical decision-making and treatment initiation.

In light of the limitations associated with conventional diagnostic methods for streptococcal pharyngitis, there has been growing interest in leveraging AI to enhance diagnostic accuracy, efficiency, and accessibility in remote or resource-limited rural Indigenous regions.

### 2.2. Why AI Is Essential but Challenging for Strep Throat Diagnosis

AI is increasingly being integrated into healthcare systems; AI supports a wide range of medical support, from drug discovery and hospital resource management to more direct clinical applications such as collaborative diagnosis and imaging analysis [15]. As medical technologies advance, Lu [16] suggested that healthcare professionals may become increasingly reliant upon them, potentially at the expense of traditional clinical competencies such as physical examination skills, patient communication, and clinical judgement.

However, Jabbour et al. [17] investigated the implications of overreliance on AI by examining how physicians and nurse practitioners responded to clinical vignettes related to pneumonia, heart failure, and chronic obstructive pulmonary disease. Participants were asked to assess the cases both independently and with the support of AI-generated diagnostic probabilities and Grad-CAM visualizations, which identify areas of the input data most influential in the AI decision-making process [18]. The results show that diagnostic accuracy can be improved when clinicians use standard and unbiased AI models, but the accuracy can be decreased when AI models contain systematic biases. These results demonstrate the potential risks associated with AI.

Moreover, the deployment of AI solutions faces unique challenges, since internet access is limited and computational resources are often constrained in rural and remote Indigenous communities. Many AI models require demanding, non-portable computing devices and reliable connectivity, which are typically unavailable in these settings. Therefore, designing lightweight, interpretable AI models that can operate effectively in resource-limited environments is essential to ensure that these communities can benefit from AI-assisted healthcare without exacerbating existing disparities. This approach not only mitigates the risk of overreliance but also fosters trust and practicality in AI-enabled diagnostics.

## 3. Proposed AI-Based Model for Strep Throat Diagnosis in Rural and Remote Indigenous Communities

Classical machine learning algorithms are still valuable due to their efficiency, transparency, and ease of interpretation. For instance, Askarian et al. [19] employed a novel approach by utilizing changes in the color space of throat images to detect strep throat. Specifically, they applied the K-nearest neighbor (KNN) algorithm to the distribution of color components within the images for classification purposes. This method capitalizes on the simplicity and interpretability of KNN, a model that does not require intensive computational resources like many deep learning frameworks. However, the reliance on specialized equipment and specific image sensor settings to capture the necessary photo qualities limits the practical applicability of this approach. The need for such specialized image sensors can constrain the method’s utility in general clinical settings, where such resources may not be readily available.

Despite classical machine learning, several studies have explored deep learning for automated strep throat diagnosis using throat images. Tobias et al. [20] first introduced a neural network-based approach for automated strep throat diagnosis. However the methodology was described at a high level; deep learning was only partially applied, and the overall system lacked rigorous empirical evaluation. Yoo et al. [6] applied transfer learning with Generative Adversarial Network (GAN)-based augmentation and achieved high accuracy; however, they did not report inference time or model complexity, and the dataset partitioning was unclear. Vamsi et al. [21] used deep learning, but similarly omitted computational metrics and relied on non-standard datasets. Additionally, dataset separation across training, validation, and testing was not clearly defined or standardized, which limits the reproducibility of the results. Notably, all three studies focused solely on classification without providing interpretability or explanations behind the model outputs. This is an important limitation for clinical decision-making, where transparency is essential. These gaps highlight the need for consistent benchmarking, efficiency reporting, well-defined datasets, and explainable AI in future clinical applications.

Since internet connectivity in rural and remote regions is generally limited and unstable, mobile medical devices carrying out demanding deep learning computation face challenges in maintaining reliable access to cloud-based real-time processing. Moreover, the high computational demands of many state-of-the-art deep learning approaches render them impractical for deployment in such resource-constrained environments. More important, these deep learning models operating as “black boxes” generally lack interpretability; they cannot offer transparent reasoning or clinically meaningful explanations for their diagnosis results. This lack of explainability limits their trustworthiness and hinders adoption by healthcare professionals, who require a clear understanding of diagnostic decisions to ensure patient safety and accountability.

**Figure 1:**
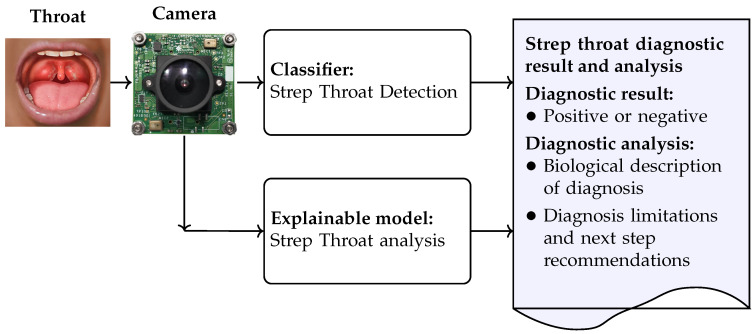
The proposed AI-based model for strep throat diagnosis and analysis.

To address the limitations of existing AI-based models for strep throat diagnosis, including high computational demands and a lack of interpretability, a strep throat diagnosis framework is proposed, as illustrated in Figure 1. The proposed AI-based model comprises two key components: a classifier for detecting strep throat and an explainable model for generating diagnostic reports and interpreting the classification results.

- **The strep throat classifier** diagnoses strep throat when a throat image is captured by the camera; the strep throat detection model was developed and its performance was evaluated in terms of detection accuracy, inference time, and model size. We attempted to find a balance between detection accuracy, model size, and inference time such that the model can be implemented on resource-limited devices, such as those available in rural and remote Indigenous communities. The development of the strep throat detection model is detailed in Section 3.1.
- **The explainable model** is used to improve understanding and transparency in strep throat diagnosis. The explainable model generates diagnostic analyses directly from throat images and produces a comprehensive report that includes both the diagnostic outcome and an explanatory narrative. The inclusion of interpretable outputs enhances clinical acceptability. In particular, this approach addresses the resistance to unfamiliar AI technologies often observed in rural and remote Indigenous communities. Both model visualization and the explainable model can be deployed to foster trust and support informed clinical decision-making. The development of the explainable model is detailed in Section 3.2.

### 3.1. Strep Throat Detection Classifier

Two streams of deep learning models, convolutional neural networks in Section 3.1.1 and vision transformer in Section 3.1.2 for strep throat detection, were considered. Their performance was evaluated in terms of detection accuracy, inference time, and computational resource requirements. The most suitable model was the one that provided a trade-off among these factors that could be selected for integration into the proposed AI-based model.

#### 3.1.1. Convolutional Neural Networks

Convolutional Neural Networks (CNNs) are optimized for image analysis; they are well-suited for medical imaging tasks. In clinical applications, transfer learning is used to adapt large, pre-trained CNNs to specific diagnostic tasks, even with limited medical datasets. These pre-trained CNNs can then be fine-tuned for downstream tasks without requiring long training times or large datasets.

In the context of strep throat detection, Yoo et al. [6] applied transfer learning using pre-trained models on the Yoo-Pharyngitis dataset. Their implementation of the ResNet-50 architecture achieved an accuracy of 95.3%. In comparison, the same dataset yielded slightly lower accuracies of 93.7% and 94.9% when using MobileNet-V2 and Inception-V3, respectively. More recently, Chng et al. [22] reported a marginally higher accuracy of 95.5% with the EfficientNetB0 model, which is a more advanced and deeper architecture.

#### 3.1.2. Vision Transformers

Vision transformers (ViTs) represent a novel adaptation of transformer architectures originally developed for Natural Language Processing (NLP) to the domain of computer vision, including medical imaging [23]. In ViTs, an image is partitioned into fixed-size patches, which are treated analogously to tokens in NLP. These image tokens are then processed using self-attention mechanisms in order to capture long-range dependencies and global contextual information.

The Convolution and Transformer Network (CoAtNet) is a hybrid architecture that combines the local feature extraction capabilities of Convolutional Neural Networks (CNNs) with the global representational power of vision transformers [24]. By integrating both paradigms, CoAtNet is well-suited for complex image classification tasks in medical imaging. Vamsi et al. [21] fine-tuned this model on the Yoo-Pharyngitis dataset for strep throat detection [6], achieving a notable accuracy of 96.6%, surpassing previous CNN- and transformer-based models.

The accuracy of the throat classification for those reviewed models is summarized in Table 2. Similar models were selected to be benchmarked in this study, including ResNet-50 [6], MobileNet V2 [6], Swin Transformer [7], VGGNet-16 [9], and ViT-B/16 [9]. Their performance was evaluated in terms of detection accuracy, inference time, and the number of parameters, as detailed in Section 4.2.

### 3.2. Explainable Models

Although the aforementioned deep learning models achieved impressive diagnostic accuracy rates of up to 97.1% in detecting strep throat, their integration into clinical practice warrants careful consideration. Reliance on opaque AI systems for strep throat diagnosis still undermines clinicians’ confidence and compromises their ability to make medical decisions. Therefore, AI-based diagnostic tools for strep throat must be implemented with safeguards that preserve professional judgment and ensure explainability.

To ensure that the AI-based model can be sustainably integrated into healthcare settings, particularly to support and empower nurses, transparency and explainability are essential. Instead of justifying whether an image indicates strep throat, the AI-based model is required to provide interpretable explanations that highlight the key visual or clinical features influencing their decisions. The model not only enhances trust but also enables less experienced clinicians to better understand the signs of infection and make informed judgments in conjunction with their own expertise.

In the proposed AI-based model, an LLM is employed to generate semantic annotations for medical images. Rather than producing full narrative descriptions, the LLM generates concise labels in order to indicate the physical signs presenting in the image. The LLM also emulates the writing style commonly found in clinical notes exchanged among nursing professionals. The LLM offers decision support while preserving the autonomy and judgment of clinical staff.

To generate semantic annotations for medical images, vision language models (VLMs) represent a relatively recent advancement in AI [25]. In 2019, VisualBERT-based BERT-like architectures were able to jointly process visual and textual inputs for tasks such as visual question answering and image–text pairing. While VisualBERT demonstrated the feasibility of integrating multimodal features, it relied on pre-extracted object detection features as input, which added significant complexity to the overall model pipeline.

In 2021, OpenAI introduced the Contrastive Language-Image Pretraining (CLIP) model [26], which is a significant advancement of vision–language models. CLIP employs separate encoders for text and images and utilizes contrastive learning to align matching image–text pairs. This architecture enabled training on a massive dataset of over 400 million image–text pairs collected from the web. Hence, CLIP is able to perform zero-shot learning generalizing to unseen categories without task-specific fine-tuning. However, CLIP reliance relies on prompt-based text embeddings; hence, it is highly sensitive to phrasing and likely to generate inconsistent outputs. Additionally, it is trained on uncurated web data, the risk of embedded biases is increased, and its reliability is insufficient for sensitive applications such as healthcare.

Although GPT-4o possesses image analysis capabilities, its cloud-based deployment poses challenges for medical applications, where data privacy, security, and the requirement of local processing are important. The medical sector requires lightweight solutions suitable for deployment on edge devices. Hence, GPT-4o is not well-suited for clinical use.

We propose the use of BLIP-2 for medical image annotation, since BLIP-2 offers high efficiency and achieves strong performance with minimal additional parameters; it is effective for local and resource-constrained healthcare settings [10]. BLIP-2 addresses the key limitations of CLIP, which is high sensitivity to prompt phrasing and a tendency to produce inconsistent outputs. Additionally, BLIP-2 can be locally implemented and is more appropriate for resource-constrained healthcare settings, unlike GPT-4o, which requires cloud-based deployment. The architecture and operational mechanism of BLIP-2 are detailed in Section 3.2.1, while the fine-tuning approach using Low-Rank Adaptation (LoRA) is described in Section 3.2.2.

#### 3.2.1. BLIP-2

BLIP-2 bridges the gap between visual and linguistic modalities in LLMs; it enables the generation and comprehension of text from image inputs with minimal additional training [10]. BLIP-2 employs a lightweight Querying Transformer (Q-former), which extracts meaningful representations from a frozen LLM decoder. The image encoder is pre-trained and images are projected into a feature space compatible with a frozen LLM decoder. This architecture enables the BLIP-2 to effectively capture and utilize visual features without the need to retrain either the image encoder or the LLM decoder. Therefore, the computational efficiency and modularity can be enhanced.

Q-former is pre-trained on 129 million image–text pairs using three key objectives: First, image–text matching enables the model to accurately associate images with their corresponding textual descriptions. Second, image–text contrastive learning trains the model to differentiate between positive and negative image–text pairs. Third, image-grounded text generation encourages the generation of text that is contextually grounded in the visual input. A fully connected layer is subsequently employed to project the output query embeddings from Q-former into a representation that aligns with the text embedding dimension of the LLM. This Q-former module offers several advantages: it is model-agnostic and thus compatible with LLMs of varying complexity; furthermore, it surpasses the performance of the dedicated vision–language model Flamingo in zero-shot visual question answering, despite utilizing 54 times fewer trainable parameters [10,27]. Therefore, BLIP-2 was implemented in the proposed AI-based model.

#### 3.2.2. Low-Rank Adaptation

To further enhance the accuracy of BLIP-2, Low-Rank Adaptation (LoRA) [28] was proposed to fine tune the model. LoRA is an efficient fine-tuning technique that reduces the computational and memory demands associated with adapting large-scale, pre-trained models. LoRA uses trainable low-rank matrices in BLIP’s attention layers; these matrices are allowed to be updated during fine-tuning while keeping the original pre-trained parameters frozen. These low-rank matrices are used as light weights, and task-specific adaptation layers encode new information with minimal memory overhead. Therefore, LoRA is suitable for resource-constrained environments.

LoRA enables the adjustment of a relatively small subset of parameters; GPU memory consumption and training time are significantly reduced. Therefore, we propose using LoRA to fine-tune BLIP-2 with streptococcal pharyngitis (strep throat) images in order to support deployment in rural and remote Indigenous communities, which often operate in low-resource settings.

## 4. Model Implementation and Validation

### 4.1. Datasets for Throat Analysis

**Figure 2:**
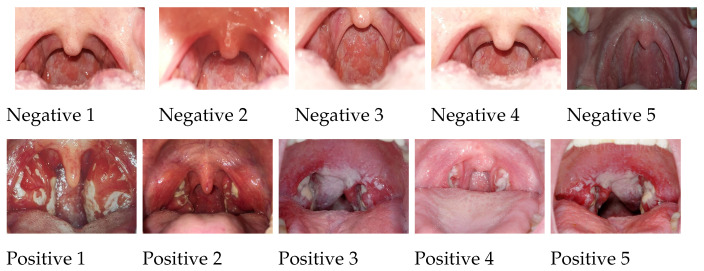
Ten images from Yoo et al. [6] depicting positive and negative cases of streptococcal pharyngitis.

To enable direct comparison, we adopted the dataset developed by Yoo et al. [6]. The dataset comprises 131 images of streptococcal pharyngitis (strep throat) and 208 images of normal throats. These images represent the only publicly available benchmark dataset for automated strep throat detection. The images were sourced from publicly available online social Q&A platforms, including Yahoo Japan and Naver Korea, as well as from search engines such as Google Images. The images cover a wide range of angles, lighting conditions, and image qualities; the dataset is robust and independent of the type of camera used for image acquisition. Classification into pharyngitis or normal categories was independently performed by two clinicians to ensure a degree of reliability in labeling. Ten images of positive and negative cases of streptococcal pharyngitis are shown in Figure 2. To further enhance the dataset for model training, augmentation and GAN-based synthesis were applied, increasing the effective sample size. This dataset was adopted primarily to evaluate classifier performance in the early stage of model development, which serves as a reproducible proof-of-concept benchmark for strep throat detection. Robust validation of Indigenous Australian communities was essential for robust validation.

Due to the small number of available samples to be used for training the classifier, data augmentation techniques were employed to expand the dataset. These included horizontal flipping, width and height translations ranging from −5% to 5%, random rotations between −10° and 10°, random zooming of up to 20%, and brightness adjustments between 90% and 110% of the original image. Such transformations enhanced the diversity of the dataset and helped the model develop invariance to minor variations. When more image data was used for the training, the generalization capability of the classifier could be improved.

To further expand the dataset, Yoo et al. [6] employed Cycle-GAN to enable image-to-image translation between two domains without requiring paired training data [29]. Cycle-GAN utilizes two generators and two discriminators, each responsible for generating and classifying images within the two domains. Training is guided by a cycle-consistency loss, which ensures that an image translated to the target domain and then back to the original domain remains consistent. By combining traditional data augmentation with Cycle-GAN, the original dataset of 169 images was expanded to 1600 images. This augmentation strategy significantly improved detection accuracy from 92.5% to 95.3%.

### 4.2. Strep Throat Detection Classifier

The models benchmarked in this study included ResNet-50 [6,30], MobileNet V2 [6,31], Swin Transformer [7,8], VGGNet-16 [9,32], and ViT-B/16 [9,33]. To conduct a comprehensive benchmarking study, the classifiers were fine-tuned to the dataset. Several performance metrics were evaluated, including detection accuracy, Receiver Operating Characteristic–Area Under the Curve (ROC-AUC) inference speed, and the number of parameters. These metrics ensured a thorough assessment of the classifier performance and its suitability for edge and real-time applications.

#### 4.2.1. Classifier Implementation

Model training was conducted in a Google Colab Pro environment using an A100 GPU. All pre-trained models were sourced from the PyTorch 2.4.0 library, with the final layer replaced by a new classifier for binary classification.

To optimize performance, a two-phase fine-tuning strategy was employed. In the first phase, all layers except the final classifier were frozen, and the new classifier was fine-tuned for 10 epochs. In the second phase, the entire model was unfrozen and trained for an additional 40 epochs. A cyclical learning rate strategy was applied, with a base learning rate of 0.0001 and a maximum of 0.001, following a triangular policy. In one cycle of the policy, the learning rate was gradually increased from the base to the maximum value and then symmetrically decreased back to the base. At the end of each cycle, the maximum learning rate was halved; this created progressively smaller triangles. This dynamic adjustment enabled the optimizer to escape shallow local minima during the rising phase and refine convergence during the falling phase. Compared with fixed or monotonically decaying learning rates, the triangular policy accelerated convergence and improved generalization while minimizing the effort of extensive hyperparameter tuning [34].

The batch size was adjusted in powers of two to maximize GPU utilization and computational efficiency. These details ensured the transparency and reproducibility of the training process.

#### 4.2.2. Detection Accuracy and ROC-AUC

The benchmarking results are presented in Table 3. The highest detection accuracy of 97.1% and an ROC-AUC of 0.993 were achieved by Swin Transformer. ViT-B/16 ranked second, achieving a detection accuracy of 89.8% and an ROC-AUC of 0.969.

To determine whether this classifier was the most suitable choice for integration into the AI-based model, additional factors such as inference time and model size were considered, since they directly impact the real-time performance of streptococcal pharyngitis detection. Evaluating inference time and model size was essential for selecting an appropriate streptococcal pharyngitis detection classifier because of the resource constraints of the AI-based model. The objective was to achieve the best trade-off between detection accuracy, inference time, and model size.

#### 4.2.3. Inference Time and Model Size

The validation results in terms of inference time and models size are detailed in Table 4. The inference time was calculated from an average of 100 inferences. The validation results show that all the classifiers achieved inference times applicable for real time applications since all inference times required by the classifiers were less than 20 ms. The fastest inference time was 1.5 ms, achieved by VGG-16. In contrast, the model size of VGG-16 was the largest. The relatively poor performance of VGG-16 in terms of model size and detection accuracy can be attributed to its homogeneous convolutional architecture and the large number of parameters. The smallest classifier was MobileNet V2, which was the most efficient for deployment on highly resource-constrained devices. Its simple structure limits its capacity to capture long-range dependencies, potentially impairing its ability to recognize spatial relationships across entire throat images.

**Figure 3:**
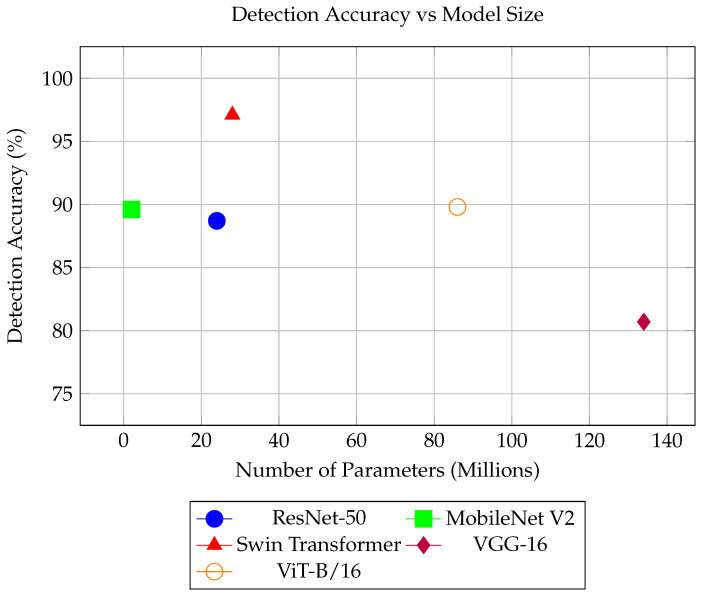
Detection accuracy vs. model size for strep throat detection models.

Figure 3 shows the detection accuracy and mobile size for each classifier. As shown in the figure, Swim Transformer offered a more advantageous trade-off than MobileNet V2 when higher detection accuracy was the primary objective. Therefore, Swim Transformer is recommended for implementation in the AI-based model for strep throat detection. The superior performance of Swim Transformer can be attributed to its ability to learn hierarchical representations [7]; it is able to capture fine-grained local details while simultaneously modeling more abstract global features.

### 4.3. Explainable Model

**Figure 4:**
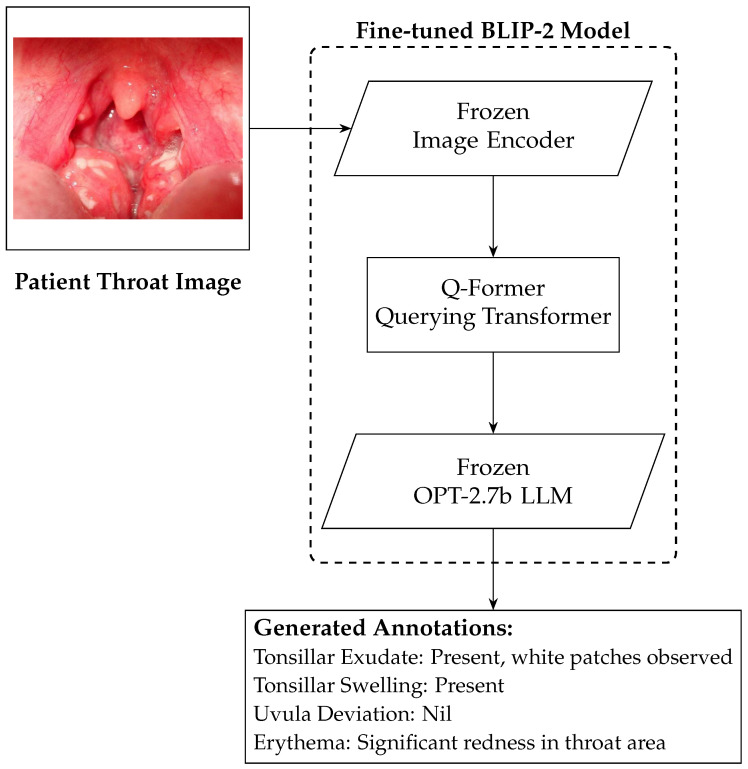
Generation of throat image annotations using BLIP-2.

While the strep throat detection model validated in Section 4.2 demonstrates acceptable performance in recognizing throat infections in terms of accuracy, inference time, and model size, the practical utility for medical practitioners remains uncertain since model predictions can be overshadowed by clinical judgments or lead practitioners to question their own expertise. To address this uncertainty, we integrated BLIP-2 (as illustrated in Figure 4) as an explainable model that elucidates the model prediction and enhances transparency. BLIP-2 ensures that the system complements rather than undermines clinical decision-making. BLIP-2 generates more semantically rich annotations and is more comparable to those found in medical reports from the same image; it has the potential to offer greater value to healthcare professionals.

The deployment of the BLIP-2 can be described in three phases: label generation to fine-tune the BLIP-2, the fine-tuning of the BLIP-2, and the validation of image annotations (see Section 4.3.1, Section 3.2.2, and Section 4.3.3 respectively).

#### 4.3.1. Label Generation

Prior to fine-tuning the BLIP-2 model, sufficient training data with throat images and semantic labels for visual signs in throat images needed to be prepared. These concise labels highlight specific signs and their locations, and estimated intensity levels are prioritized. Therefore, BLIP-2 can provide detailed descriptions of entire images; it supports nurses in confident decision-making rather than replacing clinical judgment.

For semantic label preparation, we selected GPT-4o-mini due to its strong performance in diagnostic tasks and ability to generate structured annotations from medical images [35]. Importantly, labels generated by GPT-4o-mini were used to train the smaller BLIP-2 model, which can be deployed on mobile hardware without requiring a cloud connection like GPT-4o-mini. Implementing GPT-4o-mini directly would require cloud access, which is not feasible in remote and privacy-sensitive healthcare settings. The use of GPT-4o-mini in this study is intended as a proof-of-concept; it demonstrates the feasibility of generating semantic annotations to train a light weight, and the explainable model is suitable for offline deployment. External validation against expert clinician annotations is planned in future work to ensure clinical reliability and mitigate potential biases.

**Figure 5:**
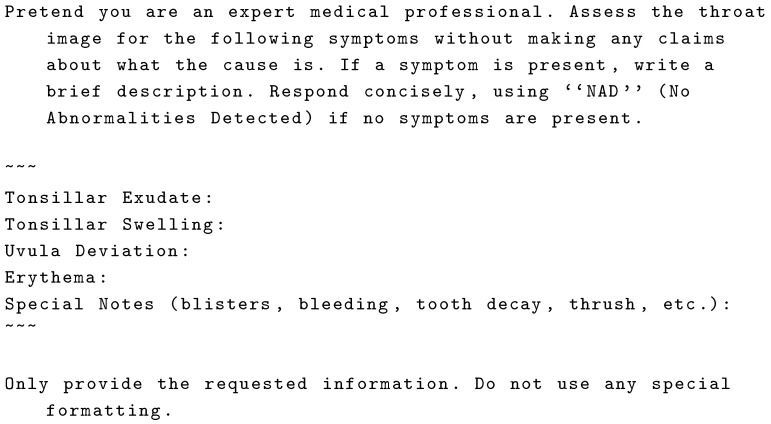
Prompt given to GPT-4o-mini along with throat image for label generation.

After trying prompt engines, we found that the best method was to directly target specific signs. The focus was narrowed to diagnostic markers regularly assessed in oral examinations, specifically targeting tonsillar exudate, tonsillar swelling, erythema, and uvular deviation [1]. This approach directs the model focus to signs with high diagnostic value in detecting strep throat; it ensures that the generated labels align closely with clinical examination practices. Utilizing the prompt illustrated in Figure 5 and the GPT-4o-mini model accessed via the OpenAI API, we generated synthetic annotations for the 200 images in the training dataset.

#### 4.3.2. Fine-Tuning

The BLIP-2 model employed in our deployment utilizes OPT-2.7b as its language backbone; it is an open-source model developed by Meta in 2022 [36]. The Open Pre-trained Transformer (OPT) series was developed to provide the research community with open access to model weights and comprehensive training methodologies; OPT serves as a competitive alternative to proprietary models such as GPT-3.5. OPT-2.7b offers a balanced trade-off between computational efficiency and performance; it is particularly suitable for resource-constrained environments, including edge deployment on platforms like the Google Colab T4 GPU. Although OPT-2.7b is a relatively small-scale model with reduced expressiveness compared to larger models (e.g., GPT-4o-mini), this limitation is acceptable within the context of our deployment, where concise and accurate responses are prioritized over creative flexibility.

In the BLIP-2 architecture, both the image encoder and the LLM were kept frozen to retain their pre-trained knowledge, while the Q-Former was fine-tuned for task-specific adaptation to align with our dataset. However, fine-tuning the Q-Former was computationally intensive. To address this, we employed Low-Rank Adaptation (LoRA) to improve training efficiency [28]. LoRA reduced the computational resources required for fine-tuning by integrating trainable low-rank matrices into the BLIP-2 model attention layers, specifically targeting the key (k_proj) and query (q_proj) projections. This approach enables task-specific customization without modifying the original pre-trained model parameters, reducing the required training memory and computational cost. In our implementation, we set the LoRA rank to 64 and the scaling factor (α) to 32 in order achieve a balance between model adaptability and resource efficiency.

The fine-tuning of the BLIP-2 model was performed on a Google Colab Pro instance equipped with an A100 GPU; we carefully selected a training dataset comprising 200 images for the fine-tuning. The model was fine-tuned over five epochs with a learning rate of 0.0005. After completing the fine-tuning, the model performance was validated with preliminary results in order to ensure the model explainability while preserving computational feasibility.

#### 4.3.3. Validation

To validate the fine-tuned BLIP-2 model, ground truth labels generated by domain experts were essential. Four resident doctors were consulted to independently examine and annotate the 110 throat images included in the validation and test sets. While the number of annotators was modest, it is important to note that in real-world clinical practice, the diagnosis of pharyngitis is typically made by a single physician’s judgment. Having consensus annotations from multiple trained doctors provided a non-trivial degree of reliability for the purpose of preliminary validation. Nonetheless, we recognize that expanding the annotator pool to include senior clinicians and specialists will be necessary in future iterations to further enhance the robustness and generalizability of the explainable model.

These expert annotations served as a reliable reference against which the GPT-4o-mini–generated labels were compared; they enabled an assessment of the accuracy of the labels that the BLIP-2 model aimed to replicate. A qualitative analysis of the results highlighted certain limitations in the generated outputs. Since OPT-2.7b is relatively compact, the responses often exhibited simplicity, repetition, and a tendency to mimic the training labels. These constraints limit the model’s capacity for nuanced and diverse observational reporting.

**Figure 6:**
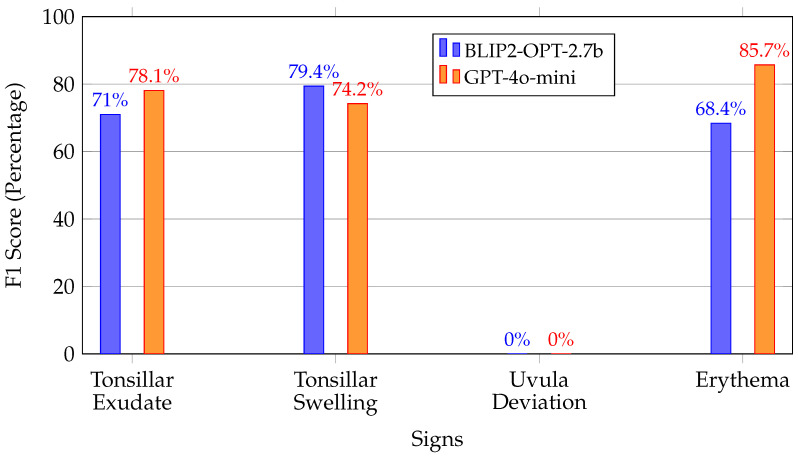
F1 scores for BLIP-2 medical sign recognition.

For quantitative evaluation, we assessed the model accuracy by comparing the signs identified in the BLIP-2 output against the ground truth annotations provided by medical professionals. Both GPT-4o-mini and the BLIP-2 model consistently failed to detect uvula deviation, uniformly reporting “NAD” (no abnormality detected) across all images. This can be attributed to the subtle nature of uvula deviation, which typically lacks prominent changes in color, texture, or size, and instead involves only a slight positional shift. This limitation underscores a potential drawback of the pipeline: biases or omissions in the original annotations are likely to be inherited by the BLIP-2 model. Figure 6 shows that the model performed reasonably well in recognizing tonsillar exudate and tonsillar swelling. However, there was a noticeable drop in performance for erythema detection, since the recognition of erythema is subjective and varies from doctor to doctor.

The validation results demonstrate that the BLIP-2 is capable of generating medical sign annotations with significant promises. BLIP-2 achieved comparable performance compared to GPT-4o-mini, which is a model with 2.7 billion parameters. Although the exact parameter count of GPT-4o-mini remains undisclosed, other large language models such as Google’s Gemini Flash—with approximately 250 billion parameters—have exhibited similar performance on the well-established MMLU benchmark [37]. These results suggest that the BLIP-2 model yields competitive results without necessitating an exponential increase in parameter size.

## 5. Conclusions

Streptococcus pharyngitis (strep throat) infections represent a serious health burden in rural and remote Indigenous communities due to their high transmissibility and potential long-term complications. The proposed AI-based model was developed to assist health practitioners in remote areas with accurate diagnosis through a dual approach: a high-accuracy classifier for strep throat detection coupled with an explainable model for assessing visible physical signs. Extensive benchmarking demonstrated that the classifier, incorporating a Swin Transformer architecture, achieved an accuracy of 97.1% and an ROC-AUC of 0.993, with an inference time of 13.8 ms and involving 28 million parameters. These metrics establish a certain level of performance for strep throat detection and also demonstrate that the classifier is feasible for edge deployment in resource-constrained settings. Furthermore, we proposed an explainable model based on BLIP-2, adapting the OPT-2.7b model for image-based reasoning with minimal computational overhead. To enhance this model, GPT-4o-mini was employed for generating synthetic data to fine-tune the BLIP-2 model. The fine-tuned model achieved an accuracy comparable to clinical expertise, as validated by medical professionals.

The current model remains in a proof-of-concept stage, where the reported accuracy and ROC-AUC demonstrate the initial feasibility. Clinician-in-the-loop studies will assess the practical utility of BLIP-2 explanations in supporting clinical decision-making. A key aspect will be testing the hypothesis that the AI system can aid nurses without fostering overreliance through studies of diagnostic performance with and without AI support [17]. Evaluating accuracy, confidence, and diagnostic speed across different support scenarios (classifier only, image annotator only, or both combined) will provide insights into optimizing AI assistance. Finally, exploring adaptations for diverse symptom patterns across communities and integration with broader diagnostic platforms will further enhance applicability and impact in rural healthcare environments.

For further validation, it is necessary to implement the model on lightweight hardware (e.g., Jetson Nano) optimized for deployment in underserved communities. This hardware-based, real-world evidence will provide additional evidence of the model’s feasibility and effectiveness in realistic clinical settings. This validation will aim to mitigate limitations related to dataset size and representativeness in order to ensure cultural and clinical relevance in future iterations of the model.

In addition, future work will focus on validating the proposed AI-based system in real-world clinical settings, in close partnership with Indigenous communities. This will include co-design with stakeholders, securing appropriate ethical approval, and ensuring cultural safety considerations that are embedded throughout the process. In addition, future technical validation is planned to extend beyond overall accuracy by including sensitivity, specificity, and false positive/negative analyses on larger, population-specific datasets in partnership with Indigenous communities. These steps will enable meaningful comparisons with established clinical benchmarks such as RADTs through prospective validation while ensuring that the system is both culturally appropriate and clinically relevant.

## Figures and Tables

**Table 1:** Overview of Clinical Prediction Rules for Strep Throat, reconstructed from Guntinas-Lichius et al. [13].

Parameter	Centor Score	McIsaac Score	FeverPAIN
Validated for	GAS	GAS	β-Hemolytic Streptococcus
Target group, age	For patients aged ≤15 years	Primarily or patient aged 3–14	
Within 3 days after onset	-	-	1 point
Body temperature > 38 °C	1 point	1 point	1 point
No cough	1 point	1 point	1 point
Cervical lymph node swelling	1 point	1 point	-
Tonsillar swelling/exudate	1 point	1 point	1 point
Tonsillar redness/inflammation	-	-	1 point
Age	-	<15 years: 1 point	-
		≥45 years: minus 1 point	
Sum of point: score and probability	0: 2.5%	0: 2.5%	0: 14%
	1: 6–7%	1: 4.4–5.7%	1: 16%
	2: 15%	2: 11%	2: 33%
	30–35%	3: 28%	3: 43%
	50–60%	4–5: 38–63%	4–5: 63%

**Table 2:** Accuracy of throat classification by reviewed papers.

Researcher	Method	Accuracy
Yoo et al. [6]	ResNet50	0.953
Chng et al. [22]	EfficientNetB0	0.955
Vamsi et al. [21]	CoAtNet	0.966

**Table 3:** Detection accuracies of strep throat classifiers.

Model	Accuracy	ROC-AUC
ResNet-50	88.7%	0.951
MobileNet V2	89.6%	0.959
Swin Transformer	97.1%	0.993
VGGNet-16	80.7%	0.909
ViT-B/16	89.8%	0.969

**Table 4:** Inference time and model sizes of strep throat detection classifiers.

Model	Inference Time (ms)	Number of Parameters
ResNet-50	6.7	24 M
MobileNet V2	5.6	2 M
Swim Transformer	13.8	28 M
VGG-16	1.5	134 M
ViT-B/16	5.3	86 M

## Data Availability

The data and implementation of methods described in this paper will be made available at https://github.com/Healthy-Connections/strep-throat-detection accessed on 31 August 2025.

## References

[1] Wiegele S., McKinnon E., van Schaijik B., Enkel S., Noonan K., Bowen A.C., Wyber R. (2023). The epidemiology of superficial Streptococcal A (impetigo and pharyngitis) infections in Australia: A systematic review. PLoS ONE.

[2] Australian Institute of Health and Welfare (2021). Acute Rheumatic Fever and Rheumatic Heart Disease in Australia, 2016–2020.

[3] Nolan-Isles D., Macniven R., Hunter K., Gwynn J., Lincoln M., Moir R., Dimitropoulos Y., Taylor D., Agius T., Finlayson H. (2021). Enablers and barriers to accessing healthcare services for Aboriginal people in New South Wales, Australia. Int. J. Environ. Res. Public Health.

[4] American Medical Association (2019). 2019 AMA Rural Health Issues Survey.

[5] Asokan P. (2024). The Role of AI in Remote Indigenous Healthcare: Detection and Annotation of Strep Throat. Bachelor’s Thesis.

[6] Yoo T.K., Choi J.Y., Jang Y., Oh E., Ryu I.H. (2020). Toward automated severe pharyngitis detection with smartphone camera using deep learning networks. Comput. Biol. Med..

[7] Liu Z., Lin Y., Cao Y., Hu H., Wei Y., Zhang Z., Lin S., Guo B. Swin Transformer: Hierarchical Vision Transformer using Shifted Windows. Proceedings of the IEEE/CVF International Conference on Computer Vision (ICCV).

[8] Cao X., Zhang Y., Lang S., Gong Y. (2023). Swin-Transformer-Based YOLOv5 for Small-Object Detection in Remote Sensing Images. Sensors.

[9] Simonyan K., Zisserman A. (2014). Very Deep Convolutional Networks for Large-Scale Image Recognition. arXiv.

[10] Li J., Li D., Savarese S., Hoi S. BLIP-2: Bootstrapping Language-Image Pre-training with Frozen Image Encoders and Large Language Models. Proceedings of the IEEE/CVF Conference on Computer Vision and Pattern Recognition (CVPR).

[11] Ashurst J.V., Edgerley-Gibb L. (2018). Streptococcal pharyngitis. StatPearls [Internet].

[12] Yeoh D.K., Anderson A., Cleland G., Bowen A.C. (2017). Are scabies and impetigo “normalised”? A cross-sectional comparative study of hospitalised children in northern Australia assessing clinical recognition and treatment of skin infections. PLoS Neglected Trop. Dis..

[13] Guntinas-Lichius O., Geißler K., Mäkitie A.A., Ronen O., Bradley P.J., Rinaldo A., Takes R.P., Ferlito A. (2023). Treatment of recurrent acute tonsillitis—A systematic review and clinical practice recommendations. Front. Surg..

[14] Willis B.H., Coomar D., Baragilly M. (2020). Comparison of Centor and McIsaac scores in primary care: A meta-analysis over multiple thresholds. Br. J. Gen. Pract..

[15] Mizna S., Arora S., Saluja P., Das G., Alanesi W. (2025). An analytic research and review of the literature on practice of artificial intelligence in healthcare. Eur. J. Med Res..

[16] Lu J. (2016). Will medical technology deskill doctors?. Int. Educ. Stud..

[17] Jabbour S., Fouhey D., Shepard S., Valley T.S., Kazerooni E.A., Banovic N., Wiens J., Sjoding M.W. (2023). Measuring the impact of AI in the diagnosis of hospitalized patients: A randomized clinical vignette survey study. JAMA.

[18] Selvaraju R.R., Cogswell M., Das A., Vedantam R., Parikh D., Batra D. Grad-cam: Visual explanations from deep networks via gradient-based localization. Proceedings of the IEEE International Conference on Computer Vision.

[19] Askarian B., Yoo S.C., Chong J.W. (2019). Novel Image Processing Method for Detecting Strep Throat (Streptococcal Pharyngitis) Using Smartphone. Sensors.

[20] Tobias R.R., De Jesus L.C., Mital M.E., Lauguico S., Bandala A., Vicerra R., Dadios E. Throat Detection and Health Classification Using Neural Network. Proceedings of the 2019 International Conference on Contemporary Computing and Informatics (IC3I).

[21] Vamsi Y., Sai Y., Dora S. (2022). Throat Infection Detection Using Deep Learning. UGC Care Group I J..

[22] Chng S.Y., Tern P.J.W., Kan M.R.X., Cheng L.T.E. (2024). Deep Learning Model and its Application for the Diagnosis of Exudative Pharyngitis. Healthc. Inform. Res..

[23] Dosovitskiy A., Beyer L., Kolesnikov A., Weissenborn D., Zhai X., Unterthiner T., Dehghani M., Minderer M., Heigold G., Gelly S. An Image is Worth 16x16 Words: Transformers for Image Recognition at Scale. Proceedings of the International Conference on Learning Representations (ICLR).

[24] Dai Z., Liu H., Le Q.V., Tan M. (2021). CoAtNet: Marrying Convolution and Attention for All Data Sizes. arXiv.

[25] Li L.H., Yatskar M., Yin D., Hsieh C.J., Chang K.W. (2019). VisualBERT: A simple and performant baseline for vision and language. arXiv.

[26] Radford A., Kim J.W., Hallacy C., Ramesh A., Goh G., Agarwal S., Sastry G., Askell A., Mishkin P., Clark J. Learning transferable visual models from natural language supervision. Proceedings of the International Conference on Machine Learning, PMLR.

[27] Alayrac J.B., Donahue J., Luc P., Miech A., Barr I., Hasson Y., Lenc K., Mensch A., Millican K., Reynolds M. (2022). Flamingo: A visual language model for few-shot learning. Adv. Neural Inf. Process. Syst..

[28] Hu E.J., Shen Y., Wallis P., Allen-Zhu Z., Li Y., Wang L., Wang W., Chen W. (2021). LoRA: Low-Rank Adaptation of Large Language Models. arXiv.

[29] Zhu J.Y., Park T., Isola P., Efros A.A. Unpaired image-to-image translation using cycle-consistent adversarial networks. Proceedings of the IEEE international Conference on Computer Vision.

[30] Cambay V.Y., Barua P.D., Baig A.H., Dogan S., Baygin M., Tuncer T., Acharya U.R. (2024). Automated Detection of Gastrointestinal Diseases Using Resnet50*-Based Explainable Deep Feature Engineering Model with Endoscopy Images. Sensors.

[31] Huang Y., Liang S., Cui T., Mu X., Luo T., Wang S., Wu G. (2024). Edge Computing and Fault Diagnosis of Rotating Machinery Based on MobileNet in Wireless Sensor Networks for Mechanical Vibration. Sensors.

[32] Perez H., Tah J.H.M., Mosavi A. (2019). Deep Learning for Detecting Building Defects Using Convolutional Neural Networks. Sensors.

[33] Nguyen H., Vo M., Hyatt J., Wang Z. (2025). AI-Powered Visual Sensors and Sensing: Where We Are and Where We Are Going. Sensors.

[34] Smith L.N. (2015). Cyclical learning rates for training neural networks. arXiv.

[35] Brin D., Sorin V., Barash Y., Konen E., Glicksberg B.S., Nadkarni G.N., Klang E. (2025). Assessing GPT-4 multimodal performance in radiological image analysis. Eur. Radiol..

[36] Zhang S., Roller S., Goyal N., Artetxe M., Chen M., Chen S., Dewan C., Diab M., Li X., Lin X.V. (2022). Opt: Open pre-trained transformer language models. arXiv.

[37] Reid M., Savinov N., Teplyashin D., Lepikhin D., Lillicrap T., Alayrac J.b., Soricut R., Lazaridou A., Firat O., Schrittwieser J. (2024). Gemini 1.5: Unlocking multimodal understanding across millions of tokens of context. arXiv.

